# High-throughput screening of monoclonal antibodies against carbapenemases using a multiplex protein microarray platform

**DOI:** 10.3389/fmicb.2025.1650094

**Published:** 2025-08-26

**Authors:** Sascha D. Braun, Martin Reinicke, Celia Diezel, Elke Müller, Katrin Frankenfeld, Thomas Schumacher, Hugo Arends, Stefan Monecke, Ralf Ehricht

**Affiliations:** ^1^Leibniz Institute of Photonic Technology, Member of the Research Alliance “Leibniz Health Technologies” and the Leibniz Centre for Photonics in Infection Research (LPI), Jena, Germany; ^2^InfectoGnostics Research Campus Jena, Center for Applied Research, Jena, Germany; ^3^INTER-ARRAY Part of fzmb GmbH, Bad Langensalza, Germany; ^4^Institut Virion\Serion GmbH, Würzburg, Germany; ^5^Institute of Physical Chemistry, Friedrich Schiller University Jena, Jena, Germany

**Keywords:** carbapenemase, protein microarray, monoclonal antibody, antimicrobial resistance, lateral flow assay, high-throughput antibody screening, point-of-care diagnostics

## Abstract

**Introduction:**

Carbapenemase-producing bacteria undermine the efficacy of carbapenems, a class of last-resort antibiotics used primarily to treat infections caused by multidrug-resistant Gram-negative pathogens. Carbapenemases are among the most alarming antimicrobial resistance mechanisms because they inactivate all β-lactam antibiotics leaving clinicians with few or no therapeutic options. The genes encoding these enzymes are typically located on mobile genetic elements (MGE), which facilitate rapid horizontal gene transfer among different bacterial species. These MGE’s often additionally carry toxin-antitoxin systems that promote long-term persistence in bacterial populations. Carbapenem-resistant *Enterobacteriaceae* (CRE) often colonize the gastrointestinal tract without symptoms, serving as silent reservoirs for further dissemination. Infections caused by CRE are associated with high morbidity and mortality and are frequently resistant to multiple drug classes. Given the urgent clinical need for rapid diagnostics, immunochromatographic assays represent a promising and urgently needed approach for economic and available point-of-care detection. However, the development of such assays is often hindered by the time-consuming process of identifying high-affinity antibody pairs.

**Methods:**

To accelerate this process, we evaluated a protein microarray platform as a high-throughput screening tool to identify optimal monoclonal antibody (mAb) pairs targeting the most clinically relevant carbapenemases. Monoclonal antibodies derived from hybridoma libraries and commercial sources were spotted in triplicates and tested in a single experiment against lysates from reference strains expressing the carbapenemase enzymes KPC, NDM, IMP, VIM, OXA-23/48/58, and MCR-1, an enzyme conferring resistance to colistin. Signal intensities were quantified, and diagnostic performance was assessed across four thresholds.

**Results:**

A cut-off > 0.2 yielded the best balance, with approximately 61% balanced accuracy and ≥99% specificity. Around 22% of tested antibodies showed strong, reproducible reactivity. For several targets–such as KPC, IMP, VIM, OXA-58, and MCR-1–100% sensitivity was achieved. The array allowed simultaneous mapping of cross-reactivity, a key advantage over conventional ELISA workflows.

**Discussion:**

Our findings confirm that protein-based microarrays offer a robust, efficient platform for antibody pair selection, reducing reagent use while accelerating assay development. The validated antibody pairs are directly applicable to ELISA or lateral flow test formats and provide a strong foundation for next-generation diagnostics capable of detecting an evolving panel of carbapenemases in clinical settings.

## 1 Introduction

Carbapenem-resistant Gram-negative bacteria are recognized as a critical threat to global health because carbapenemases inactivate almost all β-lactams and frequently act together with outer-membrane porin loss, producing pan-resistant phenotypes (van Duin and Doi, 2017; Tacconelli et al., 2018; Alvisi et al., 2025). Clinical infections caused by these organisms lead to limited therapeutic options, prolonged hospitalization and excess mortality (Perez and Bonomo, 2019), prompting their classification in the World Health Organization’s highest “critical” AMR priority tier (Govindaraj Vaithinathan and Vanitha, 2018).

The enzymatic landscape of carbapenemases is highly heterogeneous. Class A KPC, class B metallo-β-lactamases (NDM, VIM, IMP) and class D oxacillinases (OXA-48-like, OXA-23, OXA-58) account for most documented cases of carbapenem resistance, yet >1,000 allelic variants have been deposited in public databases (Alcock et al., 2020; Feldgarden et al., 2022; Gschwind et al., 2023). Many genes reside on broad-host-range plasmids equipped with toxin–antitoxin modules, ensuring stable maintenance even in absence antimicrobial pressure and facilitating rapid dissemination across *Enterobacterales*, *Acinetobacter baumannii* and *Pseudomonas aeruginosa* populations (Partridge et al., 2018; Rana et al., 2024).

Timely identification of carbapenemase producers is essential for targeted therapy and infection-control interventions, but existing diagnostics have important shortcomings. Phenotypic assays (e.g., RAPIDEC^®^ CARBA NP, ETEST^®^, or double disk synergy tests) are inexpensive but time-consuming to perform, typically requiring overnight incubation, and may miss OXA variants; nucleic-acid amplification panels provide high sensitivity but require costly instrumentation and continuous redesign to keep pace with emerging alleles; single-plex lateral-flow immunoassays detect at most five enzyme families and depend on a limited set of antibody pairs (Boutal et al., 2017). These constraints hamper large-scale surveillance and leave laboratories poorly equipped to accommodate the accelerating diversity of resistance determinants.

An ideal platform for test development should therefore (i) simultaneously screen for multiple carbapenemases, (ii) support high-throughput optimization of capture and detector monoclonal antibodies (mAbs) and (iii) generate quantitative metrics that guide transfer to user-friendly formats. Protein-based microarrays satisfy these criteria and offer several advantages over the ELISA gold standard. First, they miniaturize sandwich immunoassays into micrometer-scale spots, allowing thousands of antibody combinations to be interrogated on a single slide while using only a fraction of the reagents and sample volume required by ELISA. Second, the parallel layout exposes every candidate capture antibody to every potential detector in one experiment, enabling rapid identification of both high-affinity pairs and undesirable cross-reactivity patterns that would otherwise emerge only after weeks of sequential ELISA testing. Third, because all targets are printed side-by-side, the array provides an internal reference framework: cross-family reactivity is revealed instantly, streamlining specificity optimization. Collectively, these attributes reduce development time, lower costs and provide more detailed and quantitative insights into antibody–antigen interactions than traditional well-based methods (Kingsmore, 2006). However, protein microarrays also have limitations. They require specialized equipment for fabrication and scanning, may be sensitive to variations in antibody stability and spotting quality, and often require well-characterized monoclonal antibodies with high specificity and sensitivity. Additionally, the transition from array data to user-friendly diagnostic formats still necessitates further assay development and validation (Sauer, 2017).

Here, we evaluate a protein-based microarray comprising 49 mAbs directed against KPC, NDM, IMP, VIM, OXA-23/48/58 and the mobilized colistin-resistance factor MCR-1 (Liu et al., 2016). The specific aims were two-fold: (i) to identify high-affinity capture–detector pairs for each carbapenemase family and (ii) to quantify the diagnostic performance of the microarray across a range of signal thresholds. By establishing a streamlined discovery workflow that rapidly pinpoints optimal antibody pairs–and simultaneously unmasks cross-reactivity–this study lays the groundwork for next-generation multiplex lateral-flow or ELISA diagnostics capable of keeping pace with the evolving carbapenemase repertoire.

## 2 Materials and methods

### 2.1 Strains

Bacterial strains used in this study are listed in Table 1. The isolates were characterized by whole-genome sequencing using Oxford Nanopore Technologies (ONT) for genetic analysis and MALDI-TOF (Bruker, Bremen, Germany) for species identification. Prior to species identification, DNA extraction and long-read sequencing, all bacterial isolates were grown on Columbia blood agar plates and incubated overnight at 37 °C under aerobic conditions.

**TABLE 1:** Fully sequenced reference strains used for protein-based microarray experiments.

Number	Strain-ID	Species	Relevant resistance genes
1	CAK95354	*Klebsiella pneumoniae*	*bla*KPC-2, *bla*OXA-10
2	CAK95361	*Acinetobacter baumannii*	*bla*OXA-66, *bla*OXA-23
3	CAK95932	*Acinetobacter baumannii*	*bla*OXA-97, *bla*OXA-69
4	CAK97139	*Escherichia coli*	mcr-1
5	CAK97232	*Escherichia coli*	mcr-1
6	CAK97939	*Acinetobacter baumannii*	*bla*OXA-64, *bla*NDM-1
7	CAK97942	*Citrobacter freundii*	*bla*KPC-2
8	CAK97945	*Klebsiella pneumoniae*	*bla*IMP-8
9	CAK97963	*Klebsiella pneumoniae*	*bla*OXA-1, *bla*OXA-9, *bla*OXA-232
10	CAK97966	*Enterobacter cloacae*	*bla*OXA-2, *bla*OXA-9, *bla*OXA-163
11	CAK98019	*Citrobacter freundii*	*bla*VIM-1, *bla*VIM-2, *bla*OXA-9
12	CAK98021	*Klebsiella pneumoniae*	*bla*OXA-1, *bla*OXA-9, *bla*OXA-162
13	CAK98182	*Klebsiella pneumoniae*	*bla*OXA-9, *bla*KPC-2
14	CAK98192	*Escherichia coli*	*bla*OXA-181
15	CAK98202	*Escherichia coli*	*bla*OXA-244, *bla*NDM-1
16	CAK98224	*Escherichia coli*	*bla*OXA-244
17	CAK98887	*Acinetobacter baumannii*	*bla*OXA-23, *bla*OXA-69
18	CAK215753	*Klebsiella pneumoniae*	*bla*OXA-9, *bla*KPC-117
19	CAK215784	*Acinetobacter baumannii*	*bla*VIM-2, *bla*OXA-500
20	CAK227121	*Escherichia coli*	*bla*OXA-48
21	CAK227122	*Pseudomonas aeruginosa*	*bla*OXA-2, *bla*OXA-50, *bla*IMP-7
22	CAK227130	*Enterobacter hormachei*	*bla*IMP-14
23	CAK238636	*Klebsiella oxytoca*	*bla*VIM-1
24	CAK240608	*Escherichia coli*	*bla*OXA-48
25	CAK240609	*Acinetobacter baumannii*	*bla*OXA-69, *bla*OXA-23
26	CAK240611	*Acinetobacter baumannii*	*bla*NDM-1, *bla*OXA-64
27	CAK240614	*Escherichia coli*	*bla*IMP-1
28	CAK240615	*Escherichia coli*	*bla*OXA-9, *bla*KPC-2
29	CAK240617	*Pseudomonas aeruginosa*	*bla*IMP-1, *bla*OXA-1127
30	CAK240619	*Citrobacter freundii*	*bla*VIM-1, *bla*OXA-1, *bla*OXA-48
31	CAK240737	*Acinetobacter baumannii*	*bla*OXA-70, *bla*NDM-2
32	CAK240738	*Achromobacter xylosoxidans*	*bla*VIM-1
33	CAK240742	*Acinetobacter baumannii*	*bla*OXA-23, *bla*OXA-343, *bla*OXA-699
34	CAK240748	*Klebsiella pneumoniae*	*bla*OXA-1, *bla*KPC-2
35	CAK240749	*Klebsiella pneumoniae*	*bla*OXA-1, *bla*OXA-48, *bla*KPC-3
36	CAK240759	*Pseudomonas aeruginosa*	*bla*IMP-19, *bla*OXA-4, *bla*OXA-488
37	CAK240760	*Pseudomonas aeruginosa*	*bla*IMP-13, *bla*OXA-914
38	CAK240763	*Pseudomonas aeruginosa*	*bla*OXA-395, *bla*IMP-29, *bla*OXA-2
39	CAK240764	*Salmonella enterica*	*bla*IMP-4
40	CAK240770	*Acinetobacter baumannii*	*bla*NDM-1, *bla*OXA-69, *bla*OXA-23
41	CAK240772	*Escherichia coli*	*bla*OXA-1, *bla*NDM-1
42	CAK240774	*Escherichia coli*	*bla*NDM-4, *bla*OXA-1
43	CAK240775	*Escherichia coli*	*bla*NDM-5
44	CAK240776	*Escherichia coli*	*bla*NDM-7, *bla*OXA-1
45	CAK240780	*Escherichia coli*	*bla*VIM-4, *bla*OXA-1
46	CAK240781	*Klebsiella pneumoniae*	*bla*OXA-1, *bla*VIM-4
47	CAK240783	*Pseudomonas aeruginosa*	*bla*VIM-2, *bla*OXA-1125
48	CAK240784	*Pseudomonas putida*	*bla*VIM-5
49	CAK240785	*Acinetobacter baumannii*	*bla*OXA-66, *bla*OXA-23
50	CAK240790	*Acinetobacter baumannii*	*bla*OXA-51, *bla*OXA-58
51	CAK240799	*Klebsiella pneumoniae*	*bla*NDM-1, *bla*OXA-1, *bla*OXA-9, *bla*OXA-232
52	CAK242265	*Enterobacter cloacae*	*bla*OXA-1, *bla*NDM-1
53	CAK242268	*Citrobacter freundii*	*bla*VIM-1
54	CAK242270	*Klebsiella pneumoniae*	*bla*VIM-19
55	CAK242273	*Acinetobacter haemolyticus*	*bla*OXA-215, *bla*OXA-58
56	CAK242274	*Citrobacter freundii*	*bla*OXA-1, *bla*NDM-1, *bla*OXA-9, *bla*OXA-10
57	CAK242275	*Klebsiella pneumoniae*	*bla*OXA-1, *bla*OXA-9, *bla*OXA-58, *bla*OXA-69, *bla*OXA-162
58	CAK242814	*Klebsiella pneumoniae*	*bla*KPC-2, *bla*OXA-9
59	CAK242816	*Klebsiella pneumoniae*	*bla*VIM-2, *bla*KPC-2, *bla*OXA-9
60	CAK248610	*Escherichia coli*	*bla*OXA-1, *bla*OXA-204
61	CAK272567	*Klebsiella pneumoniae*	*bla*VIM-1
62	CAK274401	*Klebsiella pneumoniae*	*bla*KPC-2
63	CAK278788	*Acinetobacter junii*	*bla*OXA-58
64	CAK278794	*Acinetobacter lwoffii*	*bla*OXA-282
65	CAK280206	*Enterobacter cloacae*	*bla*OXA-48, *bla*NDM-5
66	CAK280207	*Pseudomonas aeruginosa*	*bla*VIM-6, *bla*OXA-10, *bla*OXA-395, *bla*OXA-1022
67	CAK280220	*Klebsiella pneumoniae*	*bla*OXA-1, *bla*OXA-9, *bla*OXA-232, *bla*NDM-1
68	CAK280609	*Klebsiella pneumoniae*	*bla*OXA-181
69	CAK280623	*Achromobacter xylosoxidans*	*bla*NDM-1, *bla*OXA-10, *bla*OXA-114f
70	CAK280640	*Klebsiella pneumoniae*	*bla*NDM-1, *bla*NDM-4
71	CAK287339	*Enterobacter kobei*	mcr-4, *bla*OXA-1
72	CAK295303	*Escherichia coli*	mcr-2, *bla*OXA-1
73	CAK296345	*Escherichia coli*	*bla*OXA-48
74	CAK296351	*Escherichia coli*	*bla*NDM-1
75	CAK301751	*Acinetobacter baumannii*	*bla*OXA-94, *bla*NDM-1, *bla*OXA-23
76	CAK303315	*Acinetobacter baumannii*	*bla*OXA-58, *bla*OXA-201
77	CAK309367	*Escherichia coli*	mcr-1
78	CAK325973	*Klebsiella pneumoniae*	mcr-1
79	CAK326361	*Klebsiella pneumoniae*	mcr-1, mcr-8

### 2.2 Sequencing and resistance genotyping

Whole-genome sequencing of all strains listed in Table 1 was performed using the Oxford Nanopore Technologies (ONT) MinION platform to confirm species identity and characterize resistance gene profiles. Genomic DNA was extracted using the NucleoSpin Microbial DNA Kit (Macherey-Nagel, Düren, Germany) with minor protocol modifications. Bacterial isolates were cultured overnight on Columbia blood agar (Becton Dickinson, Heidelberg, Germany), and biomass was collected using a full inoculation loop. Cells were suspended in 500 μL PBS (pH 7.4), pelleted by centrifugation, and resuspended in 100 μL buffer BE. Mechanical lysis was achieved using a BeatBeater (Biozym, Hessisch Oldendorf, Germany) for 5 min at maximum speed. Proteinase K digestion was followed by heat inactivation at 70 °C for 5 min. RNase A (100 mg/mL; Sigma-Aldrich, Steinheim, Germany) was then added and incubated at 37 °C for 5 min. DNA was purified and eluted in 70 μL of nuclease-free water (Carl Roth, Karlsruhe, Germany).

Library preparation was conducted using the SQK-NBD114.24 Native Barcoding Kit (Oxford Nanopore Technologies), and sequencing was performed exclusively on R10.4.1 flow cells (FLO-MIN114). DNA was size-selected using AMPure XP beads (Beckman Colter, Krefeld, Germany) at a 1:1 ratio to enrich for high-molecular-weight fragments. Sequencing runs were executed for 72 h using MinKNOW (v22.12.7), and raw signal data were recorded in POD5 format.

Basecalling was performed using Dorado (v0.9.1, Oxford Nanopore Technologies) with the high-accuracy model *res_dna_r10.4.1_e8.2_400bps_sup@2023-09-22_bacterial-methylation*. *De novo* assembly was conducted with Flye (v2.9.1-b1780), followed by four rounds of polishing with Racon (v1.5.0) using optimized parameters (match = 8, mismatch = 6, gap = 8, window = 500). Final polishing was completed with Medaka (v1.7.3) using the model *r1041_e82_400bps_bacterial_methylation*. The resulting high-quality assemblies were used to confirm resistance gene content and guide recombinant antigen selection. Resistance genotyping was performed using abricate (v1.0.0) with curated resistance gene databases (ResFinder, CARD, NCBI) to identify acquired antimicrobial resistance determinants (Supplementary File 4 Data Sheet 2.zip).

### 2.3 Antigen and antibody production

All recombinant antigens used in this study were produced and developed by our partner VIRION\SERION GmbH (Würzburg, Germany). Reference genes and protein sequences used for expression are listed in Table 2. These reference sequences were selected based on their prevalence and clinical relevance in antimicrobial resistance surveillance. All genes were cloned into Novagen’s pET-16b expression vector (Merck, Darmstadt, Germany) for recombinant protein expression. The gene sequences were codon-optimized for *Escherichia coli* when necessary, while genes of *E. coli* origin were used in their native form. Cloning was performed using *Nco*I and *Bam*HI restriction sites, ensuring precise integration into the vector. To facilitate protein purification, the expressed antigens were designed with an N-terminal 10× His-tag. The Factor Xa cleavage site present in the pET16b vector was removed to prevent unwanted proteolytic processing. The resulting construct included only the mature protein sequence, excluding signal peptides. Expression was carried out in *E. coli* BL21 (DE3) using auto-induction media to optimize yield. Protein purification was performed under native conditions via Ni-NTA affinity chromatography, followed by SDS-PAGE and Western blot analysis to confirm integrity and purity. Purified antigens were stored in PBS at a minimum concentration of 2 mg/ml. These antigens were then utilized for subsequent applications, including antibody production, microarray development, and lateral flow assay optimization.

**TABLE 2:** Resistance targets, antibiotic class, GenBank accessions, and nucleotide coordinates of the mature coding regions cloned for recombinant-antigen production.

Number	Targets	Antibiotic	Gene accession	Protein accession	Gene region
1	*bla*KPC	Carbapenem	EU447304	ACA34343	15–896
2	*bla*NDM	Carbapenem	FN396876	CAZ39946	2407–3219
3	*bla*IMP-1	Carbapenem	S71932	AAB30289	448–1188
4	*bla*VIM	Carbapenem	Y18050	CAB46686	3225–4025
5	*bla*OXA-23	Carbapenem	AJ132105	CAB69042	972–1793
6	*bla*OXA-48	Carbapenem	AY236073	AAP70012	2188–2985
7	*bla*OXA-58	Carbapenem	AY665723	AAW57529	3301–4143
8	mcr-1	Colistin	KP347127	AKF16168	22413–24038

Monoclonal antibodies were produced by our partner fzmb GmbH (Bad Langensalza, Germany) using hybridoma technology according to Köhler and Milstein (1975). Briefly, purified antigens were used to immunize mice, with multiple injections administered over several weeks to elicit a strong immune response. To enhance immunogenicity, adjuvants were included in the immunization protocol; however, the specific formulation remains undisclosed due to confidentiality agreements with the industrial partner. Finally, spleen cells were harvested and fused with immortal myeloma cells using polyethylene glycol, generating hybridoma cells capable of continuous antibody production in vitro. These hybridomas were then cultured in hypoxanthine-aminopterin-thymidine (HAT) medium to select for successfully fused cells, while unfused myeloma and B cells were eliminated. The resulting hybridomas were screened for specific antibody production using enzyme-linked immunosorbent assays (ELISA), and positive clones cloned and re-cloned. For each antigen, at least ten distinct monoclonal antibodies were generated, with each hybridoma cell clone being expanded and cultured for large-scale production. Antibodies were purified from the culture supernatant using protein A/G affinity chromatography, ensuring high purity. Finally, the purified antibodies underwent extensive characterization, including ELISA, Western blotting, and affinity determination, to confirm specificity and suitability for downstream applications such as microarray development and lateral flow assays.

Additionally, commercially available antibodies from various manufacturers were incorporated into the study to complement the panel of monoclonal antibodies generated via hybridoma technology. These antibodies were commercially obtained from different suppliers to ensure broad coverage and validated specificity for the targeted resistance determinants. These antibodies are listed in Table 3, detailing their origin, target-specificity, and order number. The inclusion of commercially available antibodies allowed for comparative validation and facilitated the development of a robust detection platform suitable for microarray and lateral flow assay applications.

**TABLE 3:** Diagnostic performance of capture antibodies using the detection antibody mix against various carbapenemase and resistance targets at increasing array signal thresholds.

					Array signal threshold > 0.1			Array signal threshold > 0.2			Array signal threshold > 0.3			Array signal threshold > 0.5		
Manu-facturer	Target	Capture-antibody	Order #	Targets tested	Spec	Sens	Accur	Spec	Sens	Accur	Spec	Sens	Accur	Spec	Sens	Accur
**Certest**	**IMP**	***bla*IMP-clone-05**	**MT-16IM05**	***bla*IMP-1**	**98.7%**	**100.0%**	**98.7%**	**100.0%**	**100.0%**	**100.0%**	**100.0%**	**100.0%**	**100.0%**	**100.0%**	**100.0%**	**100.0%**
Certest	IMP	*bla*IMP-clone-01	MT-16IM01	*bla*IMP-1	94.8%	0.0%	92.4%	94.8%	0.0%	92.4%	94.8%	0.0%	92.4%	94.8%	0.0%	92.4%
Certest	IMP	*bla*IMP-clone-18	MT-16IM18	*bla*IMP-1	100.0%	0.0%	97.5%	100.0%	0.0%	97.5%	100.0%	0.0%	97.5%	100.0%	0.0%	97.5%
Certest	KPC	*bla*KPC-clone-05	MT-16KP05	*bla*KPC-2, *bla*KPC-3, *bla*KPC-117	100.0%	0.0%	87.3%	100.0%	0.0%	87.3%	100.0%	0.0%	87.3%	100.0%	0.0%	87.3%
**Certest**	**KPC**	***bla*KPC-clone-58**	**MT-16KP58**	***bla*KPC-2, *bla*KPC-3, *bla*KPC-117**	**100.0%**	**100.0%**	**100.0%**	**100.0%**	**100.0%**	**100.0%**	**100.0%**	**100.0%**	**100.0%**	**100.0%**	**100.0%**	**100.0%**
Certest	KPC	*bla*KPC-clone-43	MT-16KP43	*bla*KPC-2, *bla*KPC-3, *bla*KPC-117	100.0%	60.0%	94.9%	100.0%	60.0%	94.9%	100.0%	60.0%	94.9%	100.0%	60.0%	94.9%
Certest	NDM	*bla*NDM-clone-22	MT-16ND22	*bla*NDM-1, *bla*NDM-2, *bla*NDM-4, *bla*NDM-5, *bla*NDM-7	98.4%	72.2%	92.4%	100.0%	61.1%	91.1%	100.0%	55.6%	89.9%	100.0%	55.6%	89.9%
Certest	NDM	*bla*NDM-clone-24	MT-16ND24	*bla*NDM-1, *bla*NDM-2, *bla*NDM-4, *bla*NDM-5, *bla*NDM-7	98.4%	27.8%	82.3%	100.0%	27.8%	83.5%	100.0%	27.8%	83.5%	100.0%	27.8%	83.5%
**Certest**	**NDM**	***bla*NDM-clone-22,** ***bla*NDM-clone-24**	**–**	***bla*NDM-1, *bla*NDM-4, *bla*NDM-5, *bla*NDM-2, *bla*NDM-7**	**98.4%**	**100.0%**	**98.7%**	**100.0%**	**88.9%**	**97.5%**	**100.0%**	**83.3%**	**96.2%**	**100.0%**	**83.3%**	**96.2%**
Certest	NDM	*bla*NDM-clone-10	MT-16ND10	*bla*NDM-1, *bla*NDM-4, *bla*NDM-5, *bla*NDM-2, *bla*NDM-7	100.0%	0.0%	77.2%	100.0%	0.0%	77.2%	100.0%	0.0%	77.2%	100.0%	0.0%	77.2%
Certest	NDM	*bla*NDM-clone-71	MT-16ND71	*bla*NDM-1, *bla*NDM-4, *bla*NDM-5, *bla*NDM-2, *bla*NDM-7	98.4%	0.0%	75.9%	100.0%	0.0%	77.2%	100.0%	0.0%	77.2%	100.0%	0.0%	77.2%
**Certest**	**VIM**	***bla*VIM-clone-20**	**MT-16VI20**	***bla*VIM-1, *bla*VIM-2, *bla*VIM-4, *bla*VIM-5, *bla*VIM-6, *bla*VIM-19**	**96.9%**	**100.0%**	**97.5%**	**96.9%**	**100.0%**	**97.5%**	**96.9%**	**100.0%**	**97.5%**	**96.9%**	**100.0%**	**97.5%**
**Certest**	**VIM**	***bla*VIM-clone-32**	**MT-16VI32**	***bla*VIM-1, *bla*VIM-2, *bla*VIM-4, *bla*VIM-5, *bla*VIM-6, *bla*VIM-19**	**95.4%**	**100.0%**	**96.2%**	**95.4%**	**100.0%**	**96.2%**	**95.4%**	**100.0%**	**96.2%**	**96.9%**	**100.0%**	**97.5%**
Certest	VIM	*bla*VIM-clone-21	MT-16VI21	*bla*VIM-1, *bla*VIM-5, *bla*VIM-2, *bla*VIM-4, *bla*VIM-19, *bla*VIM-6	100.0%	0.0%	82.3%	100.0%	0.0%	82.3%	100.0%	0.0%	82.3%	100.0%	0.0%	82.3%
FZMB	NDM	NDM_cAB01	This study	*bla*NDM-1, *bla*NDM-2, *bla*NDM-4, *bla*NDM-5, *bla*NDM-7	98.4%	16.7%	79.7%	100.0%	0.0%	77.2%	100.0%	0.0%	77.2%	100.0%	0.0%	77.2%
FZMB	NDM	NDM_cAB02	This study	*bla*NDM-1, *bla*NDM-4, *bla*NDM-5, *bla*NDM-2, *bla*NDM-7	98.4%	0.0%	75.9%	100.0%	0.0%	77.2%	100.0%	0.0%	77.2%	100.0%	0.0%	77.2%
FZMB	NDM	NDM_cAB03	This study	*bla*NDM-1, *bla*NDM-4, *bla*NDM-5, *bla*NDM-2, *bla*NDM-7	100.0%	0.0%	77.2%	100.0%	0.0%	77.2%	100.0%	0.0%	77.2%	100.0%	0.0%	77.2%
FZMB	NDM	NDM_cAB04	This study	*bla*NDM-1, *bla*NDM-4, *bla*NDM-5, *bla*NDM-2, *bla*NDM-7	100.0%	0.0%	77.2%	100.0%	0.0%	77.2%	100.0%	0.0%	77.2%	100.0%	0.0%	77.2%
FZMB	NDM	NDM_cAB05	This study	*bla*NDM-1, *bla*NDM-4, *bla*NDM-5, *bla*NDM-2, *bla*NDM-7	100.0%	0.0%	77.2%	100.0%	0.0%	77.2%	100.0%	0.0%	77.2%	100.0%	0.0%	77.2%
FZMB	NDM	NDM_cAB06	This study	*bla*NDM-1, *bla*NDM-4, *bla*NDM-5, *bla*NDM-2, *bla*NDM-7	98.4%	0.0%	75.9%	100.0%	0.0%	77.2%	100.0%	0.0%	77.2%	100.0%	0.0%	77.2%
**FZMB**	**OXA-23**	**OXA-23_cAB07**	**This study**	***bla*OXA-23-like**	**100.0%**	**100.0%**	**100.0%**	**100.0%**	**85.7%**	**98.7%**	**100.0%**	**85.7%**	**98.7%**	**100.0%**	**42.9%**	**94.9%**
**FZMB**	**OXA-23**	**OXA-23_cAB08**	**This study**	***bla*OXA-23-like**	**98.6%**	**100.0%**	**98.7%**	**100.0%**	**100.0%**	**100.0%**	**100.0%**	**85.7%**	**98.7%**	**100.0%**	**85.7%**	**98.7%**
**FZMB**	**OXA-48**	**OXA-48_cAB09**	**This study**	***bla*OXA-48-like (*bla*OXA-181, *bla*OXA-232)**	**95.1%**	**94.4%**	**94.9%**	**98.4%**	**94.4%**	**97.5%**	**98.4%**	**50.0%**	**87.3%**	**98.4%**	**50.0%**	**87.3%**
FZMB	OXA-48	OXA-48_cAB10	This study	*bla*OXA-48-like (*bla*OXA-181, *bla*OXA-232)	96.7%	50.0%	86.1%	100.0%	22.2%	82.3%	100.0%	22.2%	82.3%	100.0%	11.1%	79.7%
FZMB	OXA-48	OXA-48_cAB11	This study	*bla*OXA-48-like (*bla*OXA-181, *bla*OXA-232)	96.7%	50.0%	86.1%	100.0%	50.0%	88.6%	100.0%	33.3%	84.8%	100.0%	22.2%	82.3%
**FZMB**	**OXA-58**	**OXA-58_cAB12**	**This study**	***bla*OXA-58-like**	**100.0%**	**100.0%**	**100.0%**	**100.0%**	**100.0%**	**100.0%**	**100.0%**	**100.0%**	**100.0%**	**100.0%**	**100.0%**	**100.0%**
**FZMB**	**OXA-58**	**OXA-58_cAB13**	**This study**	***bla*OXA-58-like**	**100.0%**	**100.0%**	**100.0%**	**100.0%**	**100.0%**	**100.0%**	**100.0%**	**100.0%**	**100.0%**	**100.0%**	**100.0%**	**100.0%**
FZMB	VIM	VIM-1_cAB14	This study	*bla*VIM-1, *bla*VIM-2, *bla*VIM-4, *bla*VIM-5, *bla*VIM-6, *bla*VIM-19	100.0%	21.4%	86.1%	100.0%	7.1%	83.5%	100.0%	0.0%	82.3%	100.0%	0.0%	82.3%
FZMB	VIM	VIM-1_cAB15	This study	*bla*VIM-1, *bla*VIM-2, *bla*VIM-4, *bla*VIM-5, *bla*VIM-6, *bla*VIM-19	98.5%	7.1%	82.3%	100.0%	7.1%	83.5%	100.0%	0.0%	82.3%	100.0%	0.0%	82.3%
FZMB	VIM	VIM-1_cAB16	This study	*bla*VIM-1, *bla*VIM-5, *bla*VIM-2, *bla*VIM-4, *bla*VIM-19, *bla*VIM-6	100.0%	0.0%	82.3%	100.0%	0.0%	82.3%	100.0%	0.0%	82.3%	100.0%	0.0%	82.3%
FZMB	VIM	VIM-1_cAB17	This study	*bla*VIM-1, *bla*VIM-5, *bla*VIM-2, *bla*VIM-4, *bla*VIM-19, *bla*VIM-6	98.5%	0.0%	81.0%	100.0%	0.0%	82.3%	100.0%	0.0%	82.3%	100.0%	0.0%	82.3%
FZMB	VIM	VIM-1_cAB18	This study	*bla*VIM-1, *bla*VIM-5, *bla*VIM-2, *bla*VIM-4, *bla*VIM-19, *bla*VIM-6	96.9%	0.0%	79.7%	100.0%	0.0%	82.3%	100.0%	0.0%	82.3%	100.0%	0.0%	82.3%
FZMB	VIM	VIM-1_cAB19	This study	*bla*VIM-1, *bla*VIM-5, *bla*VIM-2, *bla*VIM-4, *bla*VIM-19, *bla*VIM-6	98.5%	0.0%	81.0%	100.0%	0.0%	82.3%	100.0%	0.0%	82.3%	100.0%	0.0%	82.3%
FZMB	VIM	VIM-1_cAB20	This study	*bla*VIM-1, *bla*VIM-5, *bla*VIM-2, *bla*VIM-4, *bla*VIM-19, *bla*VIM-6	98.5%	0.0%	81.0%	100.0%	0.0%	82.3%	100.0%	0.0%	82.3%	100.0%	0.0%	82.3%
FZMB	VIM	VIM-1_cAB21	This study	*bla*VIM-1, *bla*VIM-5, *bla*VIM-2, *bla*VIM-4, *bla*VIM-19, *bla*VIM-6	100.0%	0.0%	82.3%	100.0%	0.0%	82.3%	100.0%	0.0%	82.3%	100.0%	0.0%	82.3%
FZMB	IMP	IMP_cAB22	This study	*bla*IMP-1	100.0%	0.0%	97.5%	100.0%	0.0%	97.5%	100.0%	0.0%	97.5%	100.0%	0.0%	97.5%
FZMB	IMP	IMP_cAB23	This study	*bla*IMP-1	98.7%	0.0%	96.2%	100.0%	0.0%	97.5%	100.0%	0.0%	97.5%	100.0%	0.0%	97.5%
FZMB	IMP	IMP_cAB24	This study	*bla*IMP-1	100.0%	0.0%	97.5%	100.0%	0.0%	97.5%	100.0%	0.0%	97.5%	100.0%	0.0%	97.5%
FZMB	IMP	IMP_cAB25	This study	*bla*IMP-1	100.0%	0.0%	97.5%	100.0%	0.0%	97.5%	100.0%	0.0%	97.5%	100.0%	0.0%	97.5%
FZMB	IMP	IMP_cAB26	This study	*bla*IMP-1	100.0%	0.0%	97.5%	100.0%	0.0%	97.5%	100.0%	0.0%	97.5%	100.0%	0.0%	97.5%
FZMB	IMP	IMP_cAB27	This study	*bla*IMP-1	100.0%	0.0%	97.5%	100.0%	0.0%	97.5%	100.0%	0.0%	97.5%	100.0%	0.0%	97.5%
**FZMB**	**mcr-1**	**MCR-1_cAB28**	**This study**	**mcr-1**	**100.0%**	**100.0%**	**100.0%**	**100.0%**	**80.0%**	**98.7%**	**100.0%**	**80.0%**	**98.7%**	**100.0%**	**80.0%**	**98.7%**
FZMB	mcr-1	MCR-1_cAB29	This study	mcr-1	100.0%	0.0%	93.7%	100.0%	0.0%	93.7%	100.0%	0.0%	93.7%	100.0%	0.0%	93.7%
Raybiotech	NDM	*bla*NDM-1-clone-E2	130-10262-100	*bla*NDM-1, *bla*NDM-2, *bla*NDM-4, *bla*NDM-5, *bla*NDM-7	100.0%	61.1%	91.1%	100.0%	61.1%	91.1%	100.0%	50.0%	88.6%	100.0%	16.7%	81.0%
Raybiotech	NDM	*bla*NDM-1-clone-E9	130-10250-100	*bla*NDM-1, *bla*NDM-4, *bla*NDM-5, *bla*NDM-2, *bla*NDM-7	100.0%	0.0%	77.2%	100.0%	0.0%	77.2%	100.0%	0.0%	77.2%	100.0%	0.0%	77.2%
Raybiotech	NDM	*bla*NDM-1-clone-F2	130-10261-100	*bla*NDM-1, *bla*NDM-4, *bla*NDM-5, *bla*NDM-2, *bla*NDM-7	100.0%	0.0%	77.2%	100.0%	0.0%	77.2%	100.0%	0.0%	77.2%	100.0%	0.0%	77.2%
Raybiotech	NDM	*bla*NDM-1-clone-F3	130-10283-100	*bla*NDM-1, *bla*NDM-4, *bla*NDM-5, *bla*NDM-2, *bla*NDM-7	100.0%	0.0%	77.2%	100.0%	0.0%	77.2%	100.0%	0.0%	77.2%	100.0%	0.0%	77.2%
Raybiotech	NDM	*bla*NDM-1-clone-H3	130-10282-100	*bla*NDM-1, *bla*NDM-4, *bla*NDM-5, *bla*NDM-2, *bla*NDM-7	100.0%	0.0%	77.2%	100.0%	0.0%	77.2%	100.0%	0.0%	77.2%	100.0%	0.0%	77.2%
Raybiotech	VIM	*bla*VIM-2-clone-F3	130-10561-100	*bla*VIM-1, *bla*VIM-5, *bla*VIM-2, *bla*VIM-4, *bla*VIM-19, *bla*VIM-6	100.0%	0.0%	82.3%	100.0%	0.0%	82.3%	100.0%	0.0%	82.3%	100.0%	0.0%	82.3%
Raybiotech	VIM	*bla*VIM-2-clone-G6	130-10562-100	*bla*VIM-1, *bla*VIM-5, *bla*VIM-2, *bla*VIM-4, *bla*VIM-19, *bla*VIM-6	98.5%	0.0%	81.0%	100.0%	0.0%	82.3%	100.0%	0.0%	82.3%	100.0%	0.0%	82.3%

The table summarizes the analytical performance of multiple monoclonal antibodies targeting clinically relevant resistance determinants (e.g., IMP, KPC, NDM, VIM, OXA-23/-48/-58, and MCR-1). Each entry includes the antibody manufacturer, target group, clone name, order number (where applicable), and tested gene variants. Sensitivity (Sens), specificity (Spec), and overall accuracy (Accur) were calculated for four normalized intensity (NI) thresholds (>0.1, >0.2, >0.3, and >0.5), reflecting different cutoffs for positive signal detection. This dataset enables comparative benchmarking of candidate antibody clones for their suitability in downstream lateral flow assay (LFA) development or surveillance platforms. Resistance gene profiles confirmed by whole-genome sequencing served as the reference standard. The best-performing antibody clones, defined by consistently high sensitivity and specificity across thresholds, are highlighted in bold and shaded in gray. These candidates are considered particularly suitable for further development in rapid diagnostic formats such as lateral flow assays (LFA) or multiplex platforms for resistance surveillance.

### 2.4 Microarray production and procedure

The microarrays used in this study were manufactured by INTER-ARRAY (Part of fzmb GmbH, Research Center for Medical Technology and Biotechnology, Bad Langensalza, Germany). All antibodies were covalently immobilized directly onto functionalized plastic microarray strips (Scienion, Germany) using a fully automated M2 spotter (M2 Automation, Berlin, Germany). The spotted area measured 3.5 mm by 3.5 mm, accommodating 196 spots with a diameter of 80–120 μm (for the microarray layout, see Supplementary File 1 Table1.xlsx). The spotting process ensured uniform distribution and optimal binding conditions for each antibody, which were applied at a final concentration of 0.25 μg/μL. All antibodies were provided at 1 mg/mL by FZMB or commercial suppliers and diluted accordingly prior to spotting. Following manufacturing, each 8-well strip was sealed under an argon atmosphere to maintain stability and stored at room temperature until use. This approach preserved antibody functionality and ensured reproducibility in downstream applications.

**FIGURE 1:**
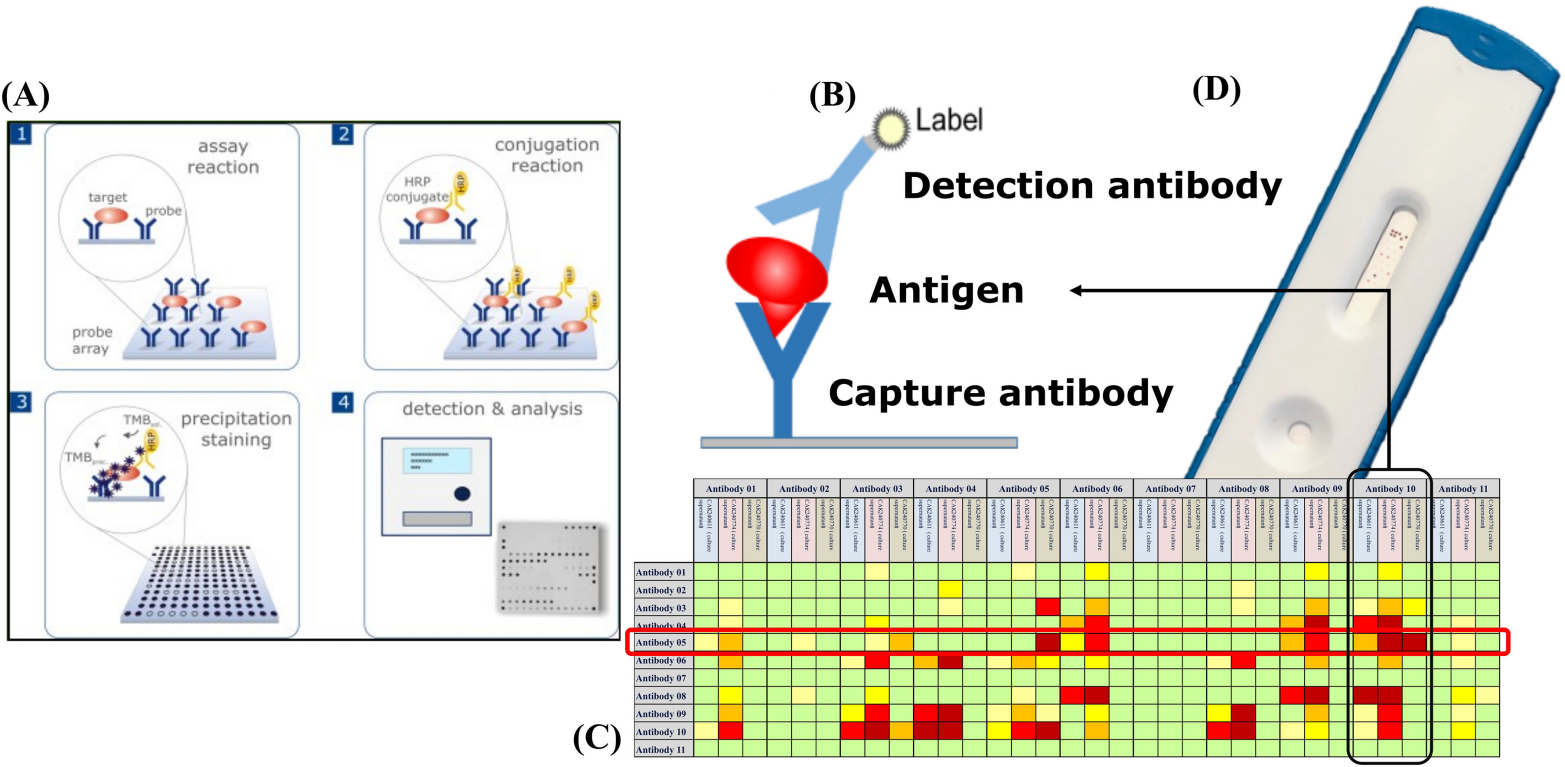
High-throughput microarray screening for perfectly matched antibody pairs: **(A)** Microarray workflow: an array of immobilized capture antibodies first binds the native antigen, after which streptavidin-horseradish peroxidase (HRP)–tagged detection antibodies are added; enzymatic conversion of the insoluble Seramun-Blue substrate (TMB) then produces a dark spot that is quantified by an automated reader. **(B)** Sandwich geometry in which the surface-bound capture antibody holds the antigen in place for recognition by the HRP-labeled detector. **(C)** Heat map ranking every possible capture-detector combination (rows versus columns) by signal intensity (green, none; yellow, moderate; red, strong), highlighting the intersection of capture Antibody 05 and detector Antibody 10 as the optimal choice for device translation. **(D)** Prototype lateral-flow strip built with the top-ranked pair; the distinct test line confirms a robust signal in the point-of-care format.

The detection of resistance-associated proteins using antibody-based microarrays was carried out according to an optimized protocol (Figure 1). For all microarray experiments described in this study, bacterial lysates were used as the antigen source. Recombinant antigens were not applied to the arrays during screening. The strains were incubated on Columbia Blood agar (BD, Germany) at 37 °C for 18–24 h. One loop of cells was inoculated directly from the agar into 300 μl buffer (1xPBS; 0.05% Tween20; 0.25% TritonX-100; 1% fetal calf serum) and vortexed. The arrays were washed twice with 150 μl buffer for 3 min at 37 °C and 400 rpm using an Eppendorf Thermomixer (Eppendorf, Germany), followed by 100 μl blocking solution (10% fetal calf serum diluted in 1xPBS; 0.05% Tween20; 0.25% TritonX-100) for 5 min at 37 °C and 300 rpm. Then 100 μl of the cell suspensions were added to the microarray strip and incubated at 37 °C and 300 rpm for 30 min. The arrays were then washed with 150 μl buffer for 5 min at 37 °C and 400 rpm. The specifically bound proteins were detected by the addition of 100 μl of an antibody-detection-mix (Table 4), including eight biotin-labeled antibodies, one for each target, and incubate at 37 °C and 300 rpm for 30 min. After a washing step 100 μl of streptavidin-horseradish peroxidase (HRP) was added and incubated for 15 min at 37 °C and 300 rpm. After two final washing steps (37 °C, 400 rpm, 3 min each), the microarrays were incubated with SeramunBlue substrate (Seramun Diagnostica GmbH, Heidesee, Germany) for exactly 10 min at 25 °C without shaking to visualize antibody-antigen interactions.

**TABLE 4:** Composition of the detection antibody mix used in the microarray assay.

Antibody	Targets	Antibiotic	Manufacturer	Specificity	Order Nr.
Anti-KPC-clone05	*bla*KPC	Carbapenem	Certest	Consensus	MT-16KP05
Anti-NDM-clone24	*bla*NDM	Carbapenem	Certest	Consensus	MT-16ND24
Anti-IMP-clone01	*bla*IMP-1	Carbapenem	Certest	Consensus	MT-16IM01
Anti-VIM-clone21	*bla*VIM	Carbapenem	Certest	Consensus	MT-16VI21
OXA-23_dAB01	*bla*OXA-23	Carbapenem	FZMB	Consensus	This study
OXA-48_dAB02	*bla*OXA-48	Carbapenem	FZMB	Consensus	This study
OXA-58_dAB03	*bla*OXA-58	Carbapenem	FZMB	Consensus	This study
MCR-1_dAB04	mcr-1	Colistin	FZMB	MCR-1	This study

This table lists all monoclonal antibodies included in the detection mix for the antibody microarray. Each antibody targets a specific resistance gene associated with carbapenem or colistin resistance. Details include target gene, antibiotic class, supplier, epitope specificity, and order number.

### 2.5 Microarray image analysis

The antibody-based microarrays were scanned and automatically analyzed using the INTER-VISION device following the manufacturer’s guidelines (INTER-ARRAY part of fzmb GmbH, Bad Langensalza, Germany). Signal intensities were quantified based on predefined spot coordinates, ensuring precise evaluation of antibody-antigen interactions.

During analysis, the relative signal intensities were calculated by determining the normalized intensity (NI) for each spot. The NI values were computed using the formula NI = 1−(M/BG), where M represents the average intensity of the spot, and BG corresponds to the intensity of the local background. This normalization method ensured that NI values ranged between 0 (no detectable signal) and 1 (maximum intensity), allowing for accurate and reproducible data interpretation.

### 2.6 Statistics

For each antibody spot, diagnostic performance was assessed across four predefined array signal thresholds: >0.1, >0.2, >0.3, and >0.5. The classification outcomes were determined by comparing the microarray-based detection signals to known resistance gene profiles. These profiles, established through whole-genome sequencing using the Oxford Nanopore MinION platform (as detailed in Section “2.3 Antigen and antibody production”), served as the reference standard for evaluating antibody signal classifications. Sensitivity was defined as TP/(TP + FN); specificity as TN/(TN + FP); accuracy as (TP + TN)/(TP + TN + FP + FN); and balanced accuracy as (sensitivity + specificity)/2, where TP = true positives, TN = true negatives, FP = false positives, and FN = false negatives. All values were expressed as percentages.

To assess the effect of signal threshold variation on diagnostic performance, the Friedman test was applied, which is appropriate for comparing multiple related samples. This test was used to evaluate differences in sensitivity, specificity, and accuracy across the four signal thresholds. *Post hoc* comparisons were performed using the Wilcoxon signed-rank test to identify statistically significant differences between threshold pairs. A *p*-value < 0.05 was considered indicative of statistical significance. All statistical analyses were conducted using Python (v3.11), employing functions from the *scipy* and *pandas* packages. The corresponding analysis script is provided as Supplementary File 2 Data Sheet 1.csv.

## 3 Results

### 3.1 Overview of antibody screening performance

Microarray-based screening revealed several high-sensitivity antibody pairs, with distinct capture-detection combinations displaying strong interactions across all signal thresholds tested. Out of the 49 antibodies analyzed, approximately 22% demonstrated consistent and reliable signal intensities across all microarray layouts, indicating their suitability for carbapenemase detection applications. Notably, multiple antibodies from Certest and FZMB exhibited robust detection sensitivity. These included Certest clones *bla*IMP-clone-05, *bla*KPC-clone-58, *bla*NDM-clone-22, *bla*NDM-clone-24, *bla*VIM-clone-20 and *bla*VIM-clone-32, as well as FZMB clones OXA-23_cAB07, OXA-23_cAB08, OXA-48_cAB09, OXA-58_cAB12, OXA-58_cAB13 and MCR-1_cAB28 all of which showed very high sensitivity and specificity across all experimental thresholds. Their stable and reproducible performance highlights their potential as reliable components for downstream diagnostic development. All raw data are available at Supplementary File 3 Table2.xlsx.

### 3.2 Performance of the antibodies on the microarray

This study assessed the sensitivity of various antibodies targeting carbapenemase genes across signal thresholds of >0.1, >0.2, >0.3, and >0.5 to evaluate their effectiveness in detecting carbapenemase-producing bacteria (Table 3). Among the antibodies tested, the following 11 pairings demonstrated the highest sensitivity across all signal thresholds, making them reliable choices for diagnostic applications. *Bla*IMP-clone-05, which targets *bla*IMP-1, achieved 100% sensitivity across all thresholds, showing excellent detection consistency and robustness at any signal level. *Bla*KPC-clone-58, specific to *bla*KPC-2, *bla*KPC-3, and *bla*KPC-117, also reached 100% sensitivity across all thresholds, underscoring its strong and consistent performance in detecting KPC-related resistance, a critical target due to the clinical significance of KPC enzymes. OXA-58_cAB12 and OXA-58_cAB13, both targeting *bla*OXA-58-like genes, also maintained 100% sensitivity across all thresholds, making them highly effective for identifying *bla*OXA-58, which is an important variant in resistance profiling.

Another good performing antibody, *bla*KPC-clone-43, targeting *bla*KPC-2, *bla*KPC-3, and *bla*KPC-117, achieved 100% sensitivity at lower thresholds (>0.1 and >0.2) but showed a slight reduction in sensitivity to 60% at higher thresholds (>0.3 and >0.5), indicating strong efficacy in early-stage detection scenarios. *Bla*VIM-clone-20, which targets a broad spectrum of *bla*VIM variants (*bla*VIM-1, *bla*VIM-2, *bla*VIM-4, *bla*VIM-5, *bla*VIM-6, and *bla*VIM-19), demonstrated 100% sensitivity across all thresholds, offering comprehensive detection capability for *bla*VIM genes, which are associated with significant antibiotic resistance challenges.

OXA-23_cAB08, targeting *bla*OXA-23-like genes, exhibited 100% sensitivity at >0.2 and >0.3 thresholds, with a high sensitivity of 85.7% at >0.5, making it highly effective in detection at moderate and high signal thresholds. *Bla*NDM-clone-22, targeting multiple *bla*NDM gene variants, achieved 72.2% sensitivity at the >0.1 threshold and demonstrated gradually decreasing sensitivity at higher thresholds, reflecting its particular utility for lower-threshold detection. *Bla*NDM-1-clone-E2, targeting *bla*NDM-1, *bla*NDM-2, *bla*NDM-4, *bla*NDM-5, and *bla*NDM-7, showed sensitivity of 61.1% at the >0.1 and >0.2 thresholds, though its sensitivity decreased at higher thresholds, suggesting its optimal application in early detection settings. Finally, *bla*NDM-clone-24, targeting various *bla*NDM variants, displayed consistent sensitivity of 27.8% across thresholds >0.2, >0.3, and >0.5, providing stable detection performance at moderate and higher signal levels.

Conversely, some antibodies exhibited significantly lower sensitivity, limiting their diagnostic utility. For instance, VIM-1_cAB14 (FZMB), specific to *bla*VIM variants, showed a maximum sensitivity of just 21.4% at >0.1, decreasing further at higher thresholds, suggesting it may not be effective for consistent *bla*VIM gene detection. *Bla*NDM-1-clone-F3 and *bla*NDM-clone-71, targeting *bla*NDM-1, *bla*NDM-4, *bla*NDM-5, *bla*NDM-2, and *bla*NDM-7, exhibited no sensitivity at any threshold beyond >0.1, indicating limited applicability in most diagnostic settings that require higher detection levels, thus offering limited utility in broader diagnostic contexts.

### 3.3 Threshold optimization

Performance metrics were calculated for four array-signal cut-offs (>0.1, >0.2, >0.3 and >0.5) (Table 5). Mean specificity was ≥99% for all thresholds, whereas mean sensitivity dropped from 25.8% at >0.1% to 18.9% at >0.5, reducing balanced accuracy from 62.4% to 59.4%. A Friedman test confirmed that threshold choice significantly affected specificity (χ^2^ = 71.2, *p* < 10^–14^), sensitivity (χ^2^ = 38.4, *p* < 10^–7^) and accuracy (χ^2^ = 8.4, *p* = 0.039). *Post hoc* Wilcoxon comparisons showed that specificity at >0.1 was significantly lower than at any higher threshold (*p* ≤ 1.6 × 10^–5^), whereas results based on thresholds >0.2, >0.3 and >0.5 did not differ from one another (*p* ≈ 0.32).

**TABLE 5:** Effect of array-signal threshold on diagnostic performance.

Threshold	Mean specificity	Mean sensitivity	Mean accuracy	Balanced accuracy
>0.1	99.05%	25.79%	88.20%	62.42%
>0.2	99.76%	22.75%	88.25%	61.26%
>0.3	99.76%	20.80%	87.85%	60.28%
>0.5	99.78%	18.92%	87.56%	59.35%

Raising the cut-off above 0.2 provided no additional gain in specificity but incurred a steady loss of sensitivity. Thus, the >0.2 threshold yielded the highest overall accuracy (88.3%) and it was the best compromise between false-positive control and true-positive yield. We therefore recommend >0.2 as the default diagnostic threshold for this microarray platform and our specific test setup; a lower cut-off (>0.1) may be chosen only when maximal analytical sensitivity is essential and a slight increase in false positive rates is acceptable.

## 4 Discussion

The rise of carbapenemase-producing organisms represents a critical global health threat, compounding the broader crisis of antimicrobial resistance (AMR). Recent global estimates suggest that antimicrobial resistance (AMR) was directly responsible for approximately 1.27 million deaths in 2019, with nearly 5 million deaths associated with drug-resistant infections (Caliskan-Aydogan and Alocilja, 2023; Murray et al., 2024). The latter figure includes cases in which AMR contributed to poor clinical outcomes or treatment failure, even if it was not the primary cause of death. Carbapenem-resistant Enterobacterales (CRE) and other carbapenemase-producing pathogens represent some of the most critical threats to public health, frequently causing severe infections that are difficult to treat due to limited therapeutic options (Hong et al., 2021). Infections by these organisms are associated with significantly higher mortality rates and prolonged hospital stays, especially when appropriate therapy is delayed (Caliskan-Aydogan and Alocilja, 2023). Rapid identification of carbapenemase producers is therefore paramount to guide timely effective therapy and implement infection control measures (Richter and Marchaim, 2017). However, diagnosing carbapenemase production remains challenging. Traditional culture-based phenotypic assays (e.g., modified Hodge test (Girlich et al., 2012), RAPIDEC^®^ CARBA NP, etc.) can be laborious and slow, while molecular tests require expensive equipment and skilled personnel (Rabaan et al., 2022). Moreover, the diversity of carbapenemase genes (KPC, NDM, VIM, OXA-variants, IMP, etc.) complicates single-test detection – no single conventional assay easily covers all variants. Indeed, recent reviews emphasize the lack of cost-effective methods to broadly screen for all carbapenemase-producing species and the ongoing need for new diagnostic tools with high sensitivity across diverse enzymes (Rabaan et al., 2022). The World Health Organization has classified CRE as critical priority pathogens, underscoring the urgent need for innovative diagnostics as well as therapeutics (World-Health-Organisation, 2017).

In response to these challenges, immunochromatographic lateral flow assays (LFAs) have emerged in the past few years as rapid, practical diagnostics for the most prevalent carbapenemases. Notably, the NG-Test CARBA 5 (NG Biotech, Guipry, France), the RESIST-5 O.K.N.V.I. (Coris BioConcept, Gembloux, Belgium) and the KarbaDia (GaDIA SA, Mothey, Switzerland) kits can each detect five common carbapenemase families (KPC, OXA-48-like, NDM, VIM, IMP) within minutes. Clinical evaluations of these assays have shown excellent performance: for example, CARBA 5 achieved ∼100% sensitivity and 98%–100% specificity compared to PCR gold-standard in multicenter studies (Boutal et al., 2018; Hong et al., 2021; Kon et al., 2021). Similarly, a four-target immunochromatographic strip (K-SeT for KPC, NDM, VIM, OXA-48) reported 99.2% sensitivity and 100% specificity when testing bacterial isolates (Greissl et al., 2019). These commercially available assays have demonstrated that antibody-based detection of carbapenemase proteins can rival molecular methods in accuracy while being faster and simpler. However, immunoassays also have limitations. First, they typically target only the most common enzyme types – emerging or rare carbapenemases (e.g., GES, OXA-23 variants) may escape detection. Second, since they rely on antigen–antibody binding, sufficient protein expression is required; in practice this often means testing from a cultured isolate or an 18–24 h enrichment step to accumulate detectable enzyme levels (Wareham et al., 2016). As a result, sensitivity can drop in complex clinical specimens. Furthermore, mutations in carbapenemase genes might alter antibody binding sites and thus compromise the binding efficacy of the antibodies used for an assay (Decousser et al., 2017), necessitating continual development of new antibodies as resistance evolves. These constraints highlight a crucial bottleneck in immunoassay development – the fast, economic and complete identification of high-affinity, specific monoclonal antibody pairs for defined sample types for each relevant carbapenemase target.

Our work addresses this bottleneck by leveraging a high-throughput protein microarray to streamline the screening of monoclonal antibody pairs. Conventional ELISA-based pairing studies require testing each capture–detection antibody combination in separate wells, a low-throughput approach that becomes impractical and costly for large antibody libraries (e.g., hundreds of candidates), and that consumes considerable amounts of antibody preparations. In contrast, antibody microarrays can miniaturize and parallelize these immunoassays, allowing thousands of interactions to be evaluated simultaneously on a single slide under the same set of reaction conditions (Garcia et al., 2007). The microarray format offers clear advantages over single-plex ELISA: it is faster, conserves precious reagents and sample, and enables massive scaling of pairwise binding experiments in one experiment (Haab et al., 2001). In our prototype, 49 antibodies (from multiple sources) were spotted in an array and tested in parallel, effectively condensing what would amount to hundreds of individual ELISA tests into a single high-throughput screening assay. This approach dramatically increases the efficiency of identifying promising antibody pairs. Indeed, antibody microarrays have shown success in other fields for multiplex protein detection and antibody profiling, demonstrating high sensitivity (pg/mL levels) and robust performance when properly optimized (Garcia et al., 2007). By printing replicates on array and using a standardized readout, we achieved a platform that is both scalable and reproducible for antibody screening. Notably, the small spot size and arrayed format mean that only microgram quantities of each antibody are needed per test – an important practical benefit when screening early-stage hybridoma supernatants or precious monoclonal antibodies.

Using this microarray prototype, we surveyed a broad panel of monoclonal antibodies (mAbs) against various carbapenemase targets and rapidly pinpointed a subset with superior performance. Approximately 22% of the 49 tested antibodies emerged as top performers, displaying consistently strong signals across all array layouts and conditions. These high-sensitivity antibodies included multiple clones against the major enzyme families. For example, antibodies against *bla*IMP (*bla*IMP-clone-05) and *bla_KPC* (e.g., *bla*KPC-clone-58) achieved 100% detection sensitivity on the array at all signal thresholds evaluated, indicating robust binding to their target epitopes. Similarly, clones targeting OXA-58 and VIM carbapenemases showed uniformly high responses–often achieving 100% sensitivity even at the strictest criteria. Notably, the combination of *bla*NDM-clone-22 and *bla*NDM-clone-24 exhibited a particularly favorable diagnostic profile, yielding both high sensitivity and specificity across thresholds, and thus represent a strong candidate pair for downstream assay development targeting *bla*NDM variants. The fact that these known high-prevalence targets (KPC, NDM, VIM, OXA variants) yielded strong antibody hits is an encouraging outcome, as it aligns with clinical priorities–suggesting that the microarray effectively identified candidate detector pairs for the enzymes most needed in diagnostics. In contrast, several antibodies failed to generate appreciable signals or responded only under the most lenient threshold conditions. The weak or absent signal observed for some antibodies may reflect low binding affinity, suboptimal antigen presentation in lysates, or batch-dependent loss of antibody activity. Additionally, structural differences between recombinant immunogens and native proteins may have contributed to reduced epitope recognition. Notably, some clones that were designed to target *blaNDM* variants exhibited minimal reactivity, with sensitivity declining from 100% at the lowest cut-off to nearly 0% at more stringent thresholds. These would likely be poor choices for any diagnostic application without further optimization. The variability in performance underscores the importance of high-throughput screening: it is difficult to predict *a priori* which monoclonal antibody pairs will work well in a sandwich assay, so empirical testing of many candidates is essential. Our results provide a filtered shortlist of the most promising capture–detector pairs for each carbapenemase target, winnowing down the pool from hundreds of antibodies to a manageable handful per target enzyme.

To translate microarray data into practical diagnostic use, we analyzed the platform’s performance in terms of sensitivity and specificity at different signal thresholds. The array outputs were expressed as normalized intensity (NI) values (0–1 scale), and we evaluated four potential cut-off criteria (>0.1, >0.2, >0.3, >0.5) for calling a spot “positive.” This analysis simulates how one might set an analytical threshold in a clinical assay to balance false negatives vs. false positives. We observed that specificity was exceptionally high across all thresholds – on average ≥ 99% even at the lowest cut-off. This indicates a low false-positive rate inherent to the microarray, likely owing to the stringent washing conditions and the requirement for both capture and detection antibody binding for a signal. Increasing the stringency of the cut-off did further reduce false positives (specificity improved from ∼99.0% at NI > 0.1% to 99.8% at NI > 0.5), and this drop between >0.1 and >0.2 was statistically significant (*p* ≪ 0.001). However, raising the threshold came at the cost of sensitivity. Mean sensitivity fell from 25.8% at >0.1% to 18.9% at >0.5, reflecting the fact that weak true signals were increasingly filtered out at higher cut-offs. As a result, the balanced accuracy of the assay actually declined slightly as the threshold grew stricter (from ∼62% at >0.1 down to ∼59% at >0.5). We determined that a moderate cut-off of NI > 0.2 provides the best compromise for this microarray platform. At >0.2, overall accuracy was highest (∼88.3%) and balanced accuracy (∼61%) was close to maximal, with specificity near 99.8% while retaining ∼23% sensitivity. In practical terms, using >0.2 as the signal threshold would minimize the number of false positives without substantially sacrificing the detection of true positives. Little additional benefit was gained by harsher criteria (no significant specificity gains beyond >0.2, per *post-hoc* tests), whereas sensitivity and true-positive yield would continue to erode. We thus recommend NI > 0.2 as an optimal cut-off for interpreting this microarray’s results, though a more lenient > 0.1 threshold could be chosen in settings where maximizing sensitivity is critical and a slight increase in false positives is tolerable. This kind of threshold tuning, enabled by high throughput data, is valuable in guiding downstream diagnostic design – it establishes the signal level that differentiates specific binding from background noise under various conditions.

While the average sensitivity of the microarray (only ∼20%–25% at best) may appear low in a diagnostic sense, it is crucial to recognize that this metric was calculated across all 49 antibodies, the majority of which were non-performers under the given set of conditions. In practice, one would not use the entire array as a clinical test; instead, the few top-performing antibody pairs would be selected and developed into a focused assay. Those pairs, as demonstrated, can individually achieve 95%–100% sensitivity for their intended targets (e.g., the anti-IMP, -KPC, -VIM clones identified showed 100% sensitivity on the array). We expect that a multiplex lateral-flow device or diagnostic ELISA built from these optimal pairs would have performance on par with existing commercial kits. For example, using the selected antibodies, a combined test strip could potentially detect KPC, NDM, VIM, IMP, and OXA-48-like enzymes with near 100% sensitivity each, analogous to the RESIST-5 and CARBA-5 products. The role of the microarray described in this study is not to serve as a final diagnostic format, but rather to enable rapid identification of antibody combinations suitable for reliable diagnostic applications. This efficient screening is especially valuable as new carbapenemase variants emerge. Rather than laboriously developing one antibody pair at a time, a library of new candidate monoclonal antibodies can be arrayed and tested against the target antigen (or panel of antigens) in parallel. The power of this approach is evidenced not only by our study but also by similar high throughput pairing efforts addressing other targets. For instance, Chabi et al. (2023) employed a LFA system to screen 84 phage-display monoclonal antibody pairs for SARS-CoV-2 nucleocapsid protein and identified an optimal pair achieving an impressive 25 pg/mL limit of detection. Such examples illustrate how massively parallel antibody screening can drastically shorten the development cycle for immunoassays, yielding ultra-sensitive pairs that might be missed by smaller-scale testing. Our protein microarray provides a comparable screening pipeline for carbapenemase diagnostics – it can quickly pinpoint antibodies with the affinity and specificity needed for sensitive detection, which can then be integrated into user-friendly formats like lateral flow strips, ELISA plates, or biosensor chips.

A limitation of our study is that carbapenemase expression levels were not directly measured. While resistance genotyping via whole-genome sequencing served as the gold standard, protein expression can vary significantly under clinical conditions. It is well established that bacterial isolates may carry carbapenemase genes but express them poorly in patient samples, leading to false negatives in protein-based assays (Banerjee and Humphries, 2017). This discrepancy can be influenced by environmental factors, such as iron availability, which has been shown to downregulate NDM expression and reduce detection sensitivity (Hamprecht et al., 2018). These findings underscore the need to validate immunoassays under physiologically relevant conditions.

It is important to emphasize that our microarray is a prototype research tool for antibody pair discovery and test development, rather than a deployable diagnostic device in itself. The current platform requires laboratory instrumentation (array reader and analysis software) and skilled operators, which would not be practical in routine clinical settings. Instead, the microarray’s value lies in its ability to efficiently screen a large pool of antibody candidates and pinpoint those with the highest diagnostic potential for further development. Once top pairs are selected, they can be produced in bulk and incorporated into conventional test formats. For example, the best capture–detector combinations from this study could next be evaluated in a sandwich ELISA or a dipstick lateral flow context, where factors like antibody orientation, membrane properties, and sample matrix effects will be optimized. The knowledge gained from the microarray (e.g., relative binding strengths, cross-reactivity profiles, optimal signal thresholds) provides a strong starting point for those optimizations. In the longer term, we envision maintaining a dynamic pipeline: as new carbapenemase variants (or entirely new resistance enzymes) arise, one could rapidly screen new monoclonal antibodies on the microarray to find those that recognize the novel epitopes. This agility is essential given the evolutionary plasticity of carbapenemase genes – a point mutation could potentially hinder an antibody’s binding, but alternative antibodies might accommodate it. Our approach allows quick modifications including the addition of new capture/detector pairs to cover such mutations. The same high-density array can also serve as a post-market-surveillance tool: newly encountered clinical isolates that trigger false-positive or false-negative signals in the deployed lateral-flow test can be re-screened against the full antibody panel in a single run, rapidly revealing whether epitope drift or cross-reactivity is responsible and guiding the introduction of updated capture/detector pairs. Additionally, while we focused on carbapenemase proteins, the general microarray strategy could be extended to cover other resistance determinants (e.g., ESBLs, AmpC β-lactamases or other *mcr* allelic variants) or even to non-enzymatic biomarkers of resistance, making it a versatile platform in the fight against AMR. Finally, as microarray fabrication and detection technologies continue to advance (with increasing automation and lower costs), it is conceivable that array-based tests could eventually find a role in clinical laboratories for multiplexed pathogen or resistance detection. For now, however, the most immediate impact of our high-throughput protein microarray is as an enabling tool in the development pipeline – accelerating the creation of next-generation carbapenemase diagnostics. In an era where rapid detection of quickly evolving superbugs is critical, such tools that bridge the gap between antibody discovery and practical point-of-care tests will be invaluable in safeguarding global health.

## 5 Conclusion

This study demonstrates that a miniaturized protein-microarray provides an efficient discovery platform for sandwich antibody pairs directed against the major carbapenemase families. Screening 49 monoclonal antibodies in a single experiment rapidly narrowed the field to fewer than ten capture–detector combinations with outstanding analytical performance. Antibodies against KPC, IMP, VIM and OXA-58 enzymes displayed 100% sensitivity at all evaluated signal thresholds, while the combined use of *bla*NDM-clone-22 and *bla*NDM-clone-24 delivered the best overall balance of sensitivity and specificity for NDM variants. Statistical optimization showed that a normalized-intensity cut-off of >0.2 maximizes accuracy without compromising the very high specificity inherent to the array. These findings confirm that high-throughput microarrays can efficiently replace serial ELISA pairing studies, saving time and reagents and enabling the systematic inclusion of emerging resistance determinants. Although the present platform is a research prototype, the identified antibody pairs are immediately transferable to lateral-flow or ELISA formats, paving the way for rapid, multiplex point-of-care tests that can detect the predominant carbapenemase enzymes in minutes. The approach is readily scalable: as new carbapenemase alleles appear, additional monoclonal antibodies can be integrated into the array and evaluated in parallel, ensuring diagnostic coverage keeps pace with evolutionary changes. In summary, the study delivers a rigorously validated shortlist of high-performance antibodies and establishes a versatile methodology for continuous diagnostic innovation against carbapenemase-mediated resistance. By accelerating assay development and fostering universal access to rapid resistance testing, this strategy ultimately supports evidence-based antimicrobial stewardship and helps safeguard the dwindling effectiveness of last-line β-lactams worldwide today.

## Data Availability

The datasets presented in this study can be found in online repositories. The names of the repository/repositories and accession number(s) can be found in the article/Supplementary material.

## Supplementary material

Supplementary File 1 Microarray spotting layout of monoclonal antibodies. This table provides the layout and positional arrangement of monoclonal antibodies (mAbs) spotted on the microarray surface used in the high-throughput screening assay. Each row represents a well position on the spotting platform, indicating the unique well ID, numerical spot identifier, antibody designation (if applicable), and the spotting concentration in μg/μL. The layout corresponds to the Resistovac array prototype (Version 5) and includes both experimental and control positions. This layout was used to generate the antibody microarrays evaluated in the study. Additionally, a representative microarray image acquired with the INTER-ARRAY reader is included. The image shows results from strain CAK240772 (*E. coli*, blaNDM-1), with marked spots highlighting both a weak signal (blaNDM-clone-71) and a strong signal (blaNDM-clone-24) targeting NDM.

Supplementary File 2 Python script for statistical analysis of signal thresholds. This file contains the full Python script used to perform the statistical evaluation of antibody microarray performance across different signal intensity thresholds (>0.1, >0.2, >0.3, and >0.5). The script reads threshold-wise specificity, sensitivity, and accuracy values from a Tables–delimited input file and applies the Friedman test to detect global differences across thresholds. Pairwise comparisons between thresholds are conducted using the Wilcoxon signed-rank test to identify statistically significant differences. Output includes test statistics and *p*-values, which were used to support conclusions regarding optimal diagnostic thresholds in the main study.

Supplementary File 3 Raw microarray signal data for all reference strains. This dataset contains the raw gray values obtained from the protein microarray experiments for all tested reference strains listed in Table 1. Each row corresponds to an individual microarray spot, detailing the capture antibody, spot ID, and signal intensity (gray value) measured across all biological replicates. The dataset includes both positive and negative control samples and serves as the primary source for calculating normalized intensities (NI) and diagnostic performance metrics (sensitivity, specificity, accuracy). These raw values underpin all subsequent statistical analyses and threshold evaluations described in the main text.

Supplementary File 4 Resistance gene profiles of reference strains. This file contains the results of resistance gene identification using the ResFinder, Card and NCBI databases as implemented in the latest version of abricate. Whole-genome assemblies of all reference strains used in this study were screened to detect acquired antimicrobial resistance genes. For each isolate, detected resistance genes, sequence identity, coverage, and associated antibiotic classes are listed. These data provide a comprehensive overview of the genotypic resistance background of the strain panel used for assay development and validation.
